# Treatment Without Cost? Effects and Side Effects of an Internet-Based Intervention for Depression: Randomized Controlled Trial

**DOI:** 10.2196/71274

**Published:** 2025-07-29

**Authors:** Anna Baumeister, Lea Schuurmans, Alina Bruhns, Steffen Moritz

**Affiliations:** 1 Department of Psychiatry and Psychotherapy, University Medical Center Hamburg-Eppendorf, Martinistr. 52, Hamburg, 20246, Germany, +49 40 741053417

**Keywords:** iCBT, adverse effects, digital interventions, implementation

## Abstract

**Background:**

Internet-based interventions for depression are increasingly integrated into health care due to their effectiveness, availability, and cost-effectiveness. However, negative effects have largely been ignored.

**Objective:**

This study aimed to evaluate both positive and negative effects of an unguided intervention.

**Methods:**

In total, 303 participants were analyzed using mixed models for repeated measures to assess changes in depressive symptoms via Beck Depression Inventory-II (primary outcome) after 12 weeks compared to waitlist controls with care as usual. Secondary endpoints included depressive symptoms (Patient Health Questionnaire-9 [PHQ-9]), self-esteem, and quality of life. Negative effects were evaluated using the positive and negative effects of psychotherapy scale for internet-based interventions (PANEPS-I). Moderation analyses were conducted to explore influential effects on treatment outcomes.

**Results:**

The intervention group showed greater reduction in depressive symptoms compared to controls, with small to medium effect sizes (*g*=0.30‐0.42) with averaged 14 logins. Although improvements in self-esteem and quality of life were not observed in intention-to-treat analyses, the completer sample indicated higher self-esteem in the intervention group. Negative effects were reported by 22% (22/100) to 68% (66/97), with the highest rates for program-related effects (eg, not addressing personal problems). No moderation effects were identified.

**Conclusions:**

The intervention effectively reduces depressive symptoms. Although negative effects were present, they did not impact treatment outcome, tentatively suggesting that overall benefits may outweigh the negative effects for users.

## Introduction

Depression is one of the leading causes of disability worldwide [1,2], globally causing 158% higher direct health care excess costs in depressed compared to nondepressed adults [3]. However, there is a worldwide treatment gap for mental disorders and depression in particular [4]. Only around half of those affected by depression receive adequate treatment [5]. Hence, low-threshold and widely available digital therapy formats are evolving. To this day, various studies on internet-based therapy programs for depression, mostly based on internet-based cognitive behavioral therapy (iCBT), have demonstrated their effectiveness in depressive symptom reduction [6] and improved quality of life [7]. In addition, internet-based interventions allow easier access due to time- and location-independent use as well as a high degree of privacy and autonomy [8]. In addition, internet-based interventions are cost-effective compared to care as usual (for a review, refer to [9]).

### iCBT in the Treatment of Depression

The advantages of internet-based interventions have led to their incorporation in national treatment guidelines for various disorders [10,11], and internet-based interventions are recommended as a treatment option for mild depressive episodes [12]. In their review of the cost-effectiveness of internet-based interventions, Mitchell et al [9] stated that such programs should be included in health care systems by policy makers. In December 2019, Germany passed the Act to Improve Healthcare Provision through Digitalization and Innovation (Digital Healthcare Act), which facilitates the prescription of digital health care applications such as iCBT programs. Health insurance now fully covers the costs for those programs that have been approved by the Federal Institute for Drugs and Medical Devices.

One of the new digital health care applications is the Novego Depression program. Novego Depression has been previously investigated in 3 studies with different patient groups [13-15]. Beiwinkel et al [13] examined the Novego program under the name HelpID in a study with a sample of 180 patients with mild to moderate depressive symptoms. The study showed a significantly greater improvement in depressive symptoms in the intervention group compared to the control group. Miegel et al [14] found a significant improvement in depressive symptoms in the framework of a randomized controlled trial with 142 chronic pain patients with depressive symptoms. Participants in the intervention group showed a significant decrease in symptom severity with a small to moderate effect size (η_p_^2^=0.04 in the intention-to-treat analysis and η_p_^2^=0.06 in the per-protocol analysis). Another study evaluating the Novego Depression program demonstrated significant improvement in depressive symptoms in a sample of 58 patients with schizophrenia with comorbid moderate to severe depressive symptoms [15]. The authors found a significantly greater decrease in depressive symptom severity after using the program compared to a control group with a medium to large effect size (η_p_²=0.18).

### Negative Effects of iCBT

Despite the large and growing body of literature demonstrating the efficacy and other advantages (such as cost-effectiveness) of iCBT for depressive disorders, there still is limited research on its negative effects. Negative effects in internet-based interventions are comparable to those in face-to-face psychotherapy [16]. Meta-analyses found deterioration rates around 6% under iCBT interventions [17,18]. Fenski et al [19] examined negative effects of an internet-based intervention beyond deterioration of symptoms. Their qualitative content analysis revealed various program- and participant-related negative effects that were reported by 8% of the participants. Another study on a self-help intervention for body-focused repetitive behavior showed high frequencies of subjective negative effects (14%‐52%; [16]). The most frequently reported negative effects were lack of positive goal orientation (52%), not addressing personal problems (49%), a feeling of time or performance pressure (21%), shame regarding using the intervention (16%), and concerns about data privacy (14%). Notably, there is not yet a clear consensus on how to define and assess negative effects in internet-based interventions. However, it seems that various kinds of negative effects do occur. Most of the reported negative effects are typically program-related, underlining the relevance and importance of assessing negative effects in internet-based interventions. Program-related effects can be resolved by revising the intervention and its content.

### Aims

The demand to implement iCBT in the health care system seems well justified in view of the confirmed effectiveness, cost-effectiveness, and low-threshold accessibility of the interventions. Yet, noneconomic costs for patients, such as experiencing negative effects, need to be considered in the evaluation of such programs before implementing them in the health care system. This study investigated the effectiveness and possible negative effects of the 12-week iCBT program Novego Depression (trial registration: German Clinical Trials Register, DRKS00027459). This randomized controlled trial compared an intervention group (IG) with a waitlist control group (WCG) regarding the reduction of depressive symptoms, improvement of self-esteem, and quality of life. In addition, negative effects and satisfaction with the program as well as treatment expectations were evaluated.

## Methods

### Design

The study was designed as a 2-arm randomized controlled trial with an IG and a WCG (with access to care as usual [CAU]) and 3 measurement time points (baseline assessment [T0], interim assessment 6 weeks after baseline [T1], and post assessment 12 weeks after baseline [T2]). All assessments took place online via the survey software Qualtrics.

### Procedure

Recruitment was carried out in collaboration with 3 medical care centers in Germany. Patients were diagnosed according to the *ICD-10* (*International Statistical Classification of Diseases, Tenth Revision*) through standardized interviews with specialists or psychotherapists. Potential participants were both patients already undergoing treatment and individuals who had only visited the medical care center for diagnostic purposes and had not yet received therapy or were waiting for it. Patients who had been diagnosed with depression and were interested in participating were given access to the baseline survey providing detailed information on the study. Upon consenting to participate, respondents were directed to the baseline questionnaire. If they did not give consent, the survey was automatically canceled. During the baseline survey, further inclusion and exclusion criteria were checked. Eligible individuals who completed the baseline survey were automatically randomly assigned to one of the 2 groups (IG or WCG) in a 1:1 ratio using an algorithm within the Qualtrics survey software. IG participants subsequently received an email containing their personal access code for the intervention program as well as instructions on how to register for the program. Participants in both study groups had unrestricted access to CAU during the study period. After 6 and 12 weeks, all participants received invitations to participate in the interim and postsurveys via email with the link to the online assessment. Upon completion of the postassessment, all participants received compensation for their participation in the form of a €10 (US $11.48) online shopping voucher. Participants in the control group additionally received an access code to the intervention program. The first patient was recruited on January 6, 2022, and the last patient finished on June 6, 2023. In total, 311 participants were randomized.

### Participants

The study protocol originally envisaged the recruitment of 200 participants. In the course of the study, a new sample size calculation was performed to take into account a probable dropout rate of 20% and to gain more statistical certainty. Based on an assumed effect of *d*=0.4, an alpha of 5%, and a power of 80%, the sample size calculation was carried out on the reduced effect size of *d*=*d*x(1‐0.2)=0.32 using G*Power. This resulted in a number of cases of N=310, or n=155 per arm. The recruitment and the study procedure did not change and remained in line with the protocol. Criteria for inclusion were (1) an *ICD-10* diagnosis on the depressive spectrum without psychotic symptoms, (2) at least mild depressive symptoms at baseline (PHQ-9 Score >4), (3) age between 18 and 75 years, (4) informed consent to participate in the study, (5) access to the internet, (6) sufficient knowledge of the German language, (7) willingness to participate, and (8) a stable treatment situation (pharmacotherapy or psychotherapy or waiting for treatment, ie, no active therapy) for at least 4 weeks before randomization. Individuals were excluded from participating if (1) a psychotic or bipolar disorder had been diagnosed (lifetime), (2) acute suicidal behavior had occurred within the past 4 weeks, (3) dementia was present, (4) alcohol or substance dependence within the last 6 months had been diagnosed, (5) antipsychotics were being taken currently, or (6) a serious illness was present that would preclude the patient’s participation in the study until after the end of the study.

### Intervention

The web-based program Novego is based on evidence-based methods of CBT as well as systemic therapy and mindfulness-based therapy.

The content of the program is based on the German national guidelines for depression [20]. In addition to imparting knowledge about the disorder (psychoeducation), the program implements the 4 elementary components of CBT based on Hautzinger [21]. Thus, the content of individual modules focuses on overcoming inactivity or one-sided stressful activity through activity lists and reinforcement plans. In addition, role plays lead to improvements in social behavior, mindfulness exercises, and concentration strategies improve dysfunctional attitudes, and relapse prevention techniques enable the development of coping skills [21]. The integration of CBT third-wave approaches such as mindfulness exercises complements the program [22]. The program consists of 12 weekly modules that are automatically compiled from a total of 16 available modules based on the user’s responses to an initial in-program questionnaire. The content and images are also tailored to the personal situation of the participants. The composition is determined based on information about, for example, activity (eg, less active participants will receive Module 4a, “Discovering Activity,” whereas more active participants will receive Module 4b, “Self-Discovery”), and comorbidities such as heart disease or chronic pain. In addition, the communication with participants is tailored to their expectations of the program, and examples are adjusted based on participants’ age and gender. Each module takes approximately 45 to 60 minutes to complete. The modules include psychoeducational texts, audio files, videos, exercises, illustrations, and pictures, as well as several diary templates. In addition, users can choose to receive motivational emails and text message reminders. The entire program is made available to participants for one year after the initial 12-week period ends. Many working materials, documents, and exercises can be downloaded for long-term use. The program is web-based; installing an app is not necessary, and the program can be used on any device with an internet browser.

### Measures

#### Primary Outcome

The primary endpoint to assess efficacy of the intervention was defined as the change in severity of depressive symptoms from T0 to T2, as measured by the Beck Depression Inventory II (BDI-II; [23). The BDI-II is a valid and reliable tool for evaluating depression symptoms [24,25]. The questionnaire consists of 21 items rated on a 4-point Likert scale from 0 to 3. A sum score is calculated to assess symptom severity in the previous 2 weeks. The total score ranges from 0 to 63, with higher scores indicating a greater impairment (0‐8 no depression, 9‐13 minimal depression, 14‐19 mild depression, 20‐28 moderate depression, and 29‐63 severe depression). The BDI-II was assessed at T0, T1, and T2.

#### Secondary Outcomes

##### Patient Health Questionnaire-9

The Patient Health Questionnaire-9 (PHQ-9) [26,27] assesses the severity of depression in a self-report questionnaire covering the past 2 weeks, with 9 items answered on a 4-point Likert scale. The total score ranges from 0 to 27, with higher values indicating higher levels of depression severity. Categorization is based on the following: none or minimal (0‐4), mild (5–9), moderate (10–14), and severe (15–27) depression. The PHQ-9 has high internal consistency (Cronbach α=0.86 to 0.89; [27]). The PHQ-9 was assessed at T0, T1, and T2.

##### Rosenberg Self-Esteem Scale

The Rosenberg Self-Esteem Scale (RSES) [28] assesses self-esteem through self-reporting using 10 items answered on a 4-point Likert scale ranging from (1) “strongly agree” to (4) “strongly disagree.” The expression of self-esteem is determined by the total score, which can range from 10 to 40. A higher score indicates a stronger sense of self-esteem. The internal consistency of the scale (Cronbach *α*=0.77 to 0.88) is considered good [28]. The RSES was assessed at T0 and T2.

##### World Health Organization Quality of Life–BREF

The World Health Organization Quality of Life–BREF (WHOQOL-BREF) [29] measures subjective quality of life using 26 items. It serves as a shortened version of the WHOQOL-100 questionnaire and consists of 4 subscales (psychological well-being, physical well-being, social relationships, and environment). The response format is a 5-point Likert scale (1 to 5). For each of the 4 subscales, mean scores can be calculated, with higher values indicating better quality. Across all items, a summary score (subscale mean–4)×(100/16) between 0 and 100, with higher scores indicating better quality of life. The internal consistency (Cronbach α ranging from 0.68 to 0.82 for the four subscales) is between acceptable and good [30]. The WHOQOL-BREF was assessed at T0 and T2.

##### Positive and Negative Effects of Psychotherapy Scale for Internet-Based Interventions

The Positive and Negative Effects of Psychotherapy Scale for Internet-Based Interventions (PANEPS-I) [16] is a self-report instrument to assess unintended positive and negative effects of digital psychotherapeutic intervention. It consists of 27 items on 4 subscales: positive effects (8 items), side effects (7 items), malpractice (11 items), and unethical procedure (3 items). Responses are given on a Likert scale ranging from 1 (“agree”) to 4 (“disagree”) plus a fifth category of 5 “not applicable.” The PANEPS-I was assessed only in the IG, at T2.

##### Treatment Expectation Questionnaire

The Treatment Expectation Questionnaire (TEX-Q [31]) is a 15-item questionnaire that measures a patient’s self-reported expectations of an upcoming treatment. The questionnaire consists of 6 subscales, for each of which a mean value can be calculated: symptom improvement (items 1 to 3), positive effects (items 4 to 6), side effects (items 7 to 9), negative effects (items 10 and 11), process (items 12 and 13), and behavioral control (items 14 and 15). The items are answered on an 11-point Likert scale (0 to 10), where 0 corresponds to no expectation and 10 corresponds to the greatest conceivable expectation. If the items of the negative subscales (items 7 to 11) are inverted, an overall mean value can be calculated in which higher values represent a greater positive treatment expectation. The TEX-Q was assessed in both groups at T0.

##### Client Satisfaction Questionnaire

The Client Satisfaction Questionnaire (CSQ-8 [32]) was used to assess participants’ satisfaction with the intervention. The 8 items are rated on a 4-point Likert scale, and a sum score is calculated. Negative items are recoded so that a higher score represents higher satisfaction. The CSQ-8 has demonstrated strong validity and reliability (Cronbach α ranging from 0.87 to 0.92; [32]). The CSQ-8 was assessed in the IG at T2.

### Statistical Analyses

Statistical analyses were conducted using IBM SPSS Statistics 27 and R version 4.3.2. Differences in baseline measurements between the WCG and IG were examined using *t* tests for independent samples and chi-square tests.

### Efficacy

Mixed-effects models for repeated measures (MMRM) as well as regression analyses with multiple imputation (MI) were used to evaluate efficacy of the program in the intention-to-treat (ITT) sample. The MI analysis used the iterative Markov Chain Monte Carlo method with 10 iterations. For each iteration and variable with missing values, the method fitted a univariate model using all the other variables in the model as predictors and then imputed the missing values for the variable being fitted. The method continued until the maximum number of iterations was reached. Group allocation, age, and education level were included in the model as predictors for imputation, with 100 imputations performed.

To test for sensitivity, complete case (CC) and per protocol (PP) analyses using analysis of covariance (ANCOVA) were conducted. The CC sample included data from participants with complete datasets, and the PP sample included data from participants with complete datasets; for IG participants, the sample also included those who logged into the program at least 4 times. For the ANCOVA, the dependent variables were the differences between T0 and T2. The baseline values were included as covariates in the analysis to control for regression to the mean.

The effect sizes of the analyses were calculated using Hedges *g* (*g*≈0.2: small effect, *g*≈0.5: medium effect, and *g*≈0.8: large effect) and partial eta-squared (η_p_^2^=0.01: small, η_p_^2^=0.06: medium, η_p_^2^=0.14: large). In addition, mean differences (least squares mean differences [LSMD]) were calculated.

### Negative Effects

Negative effects were analyzed descriptively. Frequencies of each negative effect as assessed with the PANEPS-I were reported in valid percentages. In addition, the mean and SD of the sum of reported effects per subscale were calculated.

### Moderation Analyses

To identify potential variables that impacted the effect, moderation analyses were conducted using the SPSS macro PROCESS (developed by Andrew F Hayes). The exploratory endpoints (mean score of TEX-Q, sum of reported effects for each PANEPS-I subscales, and sum score of CSQ-8) and severity according to PHQ-9 categories (minimal, mild, moderate, and severe) were included as independent variables. The dependent variable was the primary endpoint (difference scores of BDI-II sum score T2–T0).

### Ethical Considerations

The study was approved by the Local Psychological Ethics Committee of the University Medical Center Hamburg-Eppendorf (ID: LPEK-0405). All participants provided informed consent. Participants were free to withdraw from the study at any time. Data were anonymized for data analysis.

## Results

### Overview

**Figure 1.**
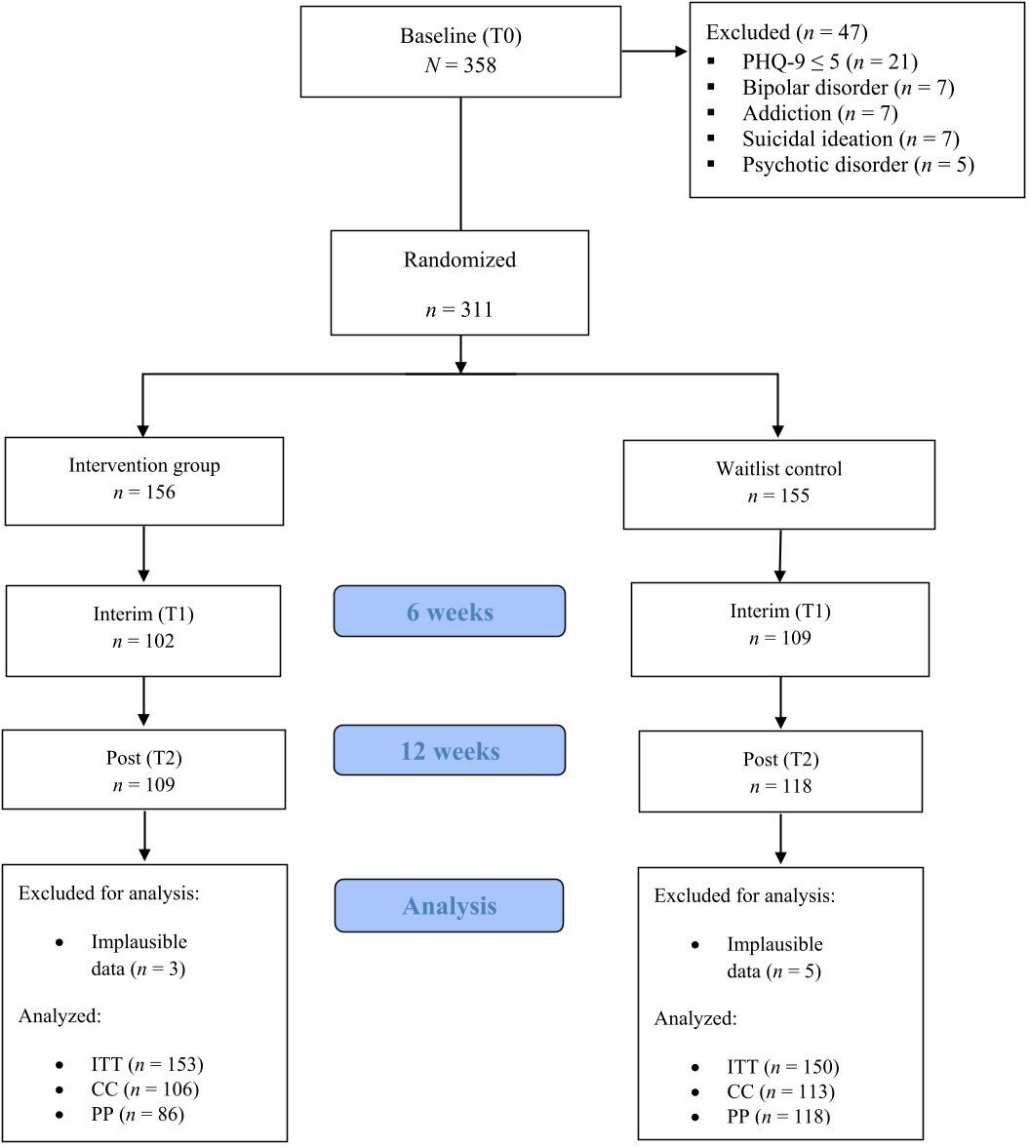
Flow chart.

A total of 358 individuals participated in the baseline survey. However, 47 individuals were excluded at T0 as they did not meet the inclusion criteria (21 individuals with a PHQ-9 score below 5) or met an exclusion criterion (7 individuals with bipolar disorder, 5 individuals with psychotic disorder, 7 individuals with substance dependence, and 7 individuals with suicidal ideation). Thus, 311 participants were randomized. The included participants were randomly assigned to either the IG (*n*=156) or the WCG (*n*=155). Eight additional participants (5 from the WCG and 3 from the IG) were excluded post hoc due to implausible responses. A result was considered implausible if the *z* score standardized T2 scores of the 2 depression measures BDI-II and PHQ-9 deviated from each other by at least 1.5 SD and if the change in the BDI-II deviated from the mean change by at least 1 SD. These participants showed contrasting courses, with improvements in one and deteriorations in the other depression scale. The final analysis thus included 303 participants (see Figure 1).

### Sample

The sample was predominantly females (218/303, 71.9%), with an average age of 40.68 (SD 12.50) years and a relatively high level of education (188/303, 62.0% at least with university entrance qualification; see Table 1). The psychiatric evaluation of the participants at baseline demonstrated an average level of depressive symptom burden (BDI-II, PHQ-9), a moderate level of self-esteem (RSES), and a moderate level of overall quality of life (WHOQOL-BREF global score; see Table 1). The accompanying CAU was well balanced among the total sample, with 47% (143/303) of participants not being in psychotherapeutic treatment at the time of baseline assessment and 41% (125/303) of the sample taking medication.

**Table 1.** Frequencies (percentages), means (SDs) of the sociodemographic and psychopathological variables at baseline.

Variable	Total (*N*=303)	WCG (*n*=150)	IG (*n*=153)	Statistics
Demographic characteristics				
Gender				*χ*²_2_=0.65, *P*=.723
Female	218 (71.9)	111 (74.0)	107 (69.9)
Male	81 (26.7)	37 (24.7)	44 (28.8)
Divers	4 (1.3)	2 (1.3)	2 (1.3)
Age in years, mean (SD)	40.68 (12.50)	40.54 (13.11)	40.82 (11.91)	*t*_299_=0.20, *P*=.843
Secondary education (at least university entrance qualification), *n* (%)	188 (62.0)	88 (58.7)	100 (65.4)	*χ*²_4_=4.19, *P*=.395
Psychopathology, mean (SD)				
BDI-II^a^	26.47 (9.52)	25.73 (10.08)	27.20 (8.68)	*t*_301_=1.36, *P*=.175
PHQ-9^b^	13.53 (4.99)	13.03 (5.23)	14.03 (4.72)	*t*_301_=1.73, *P*=.084
RSES^c^	14.19 (6.69)	14.53 (6.72)	13.86 (6.67)	*t*_301_=0.87, *P*=.387
WHOQOL^d^	39.56 (19.24)	41.42 (18.95)	37.75 (19.42)	*t*_301_=1.67, *P*=.097
Psychotherapy, *n* (%)				*χ*²_4_=6.32, *P*=.176
None	143 (47.2)	76 (50.7)	67 (43.8)	
Partial inpatient	2 (0.7)	0 (0.0)	2 (1.3)	
Inpatient	2 (0.7)	1 (0.7)	1 (0.7)	
Outpatient	135 (44.6)	67 (44.7)	68 (44.4)	
Other	21 (6.9)	6 (4.0)	15 (9.8)	
Medication *n* (%)				*χ*²_1_=4.53, *P*=.033
No intake	178 (58.7)	79 (52.7)	99 (64.7)	
Regular intake	125 (41.3)	71 (47.3)	54 (35.3)	
*Other*				
TEX-Q^e^	6.33 (1.09)	6.30 (1.00)	6.37 (1.17)	*t*_301_=0.59, *P*=.559

a BDI-II: Beck Depression Inventory-II.

b PHQ-9: Patient Health Questionnaire.

c RSES: Rosenberg Self-Esteem Scale.

d WHOQOL: World Health Organization Quality of Life Short Questionnaire.

e TEX-Q: Treatment Expectation Questionnaire.

Overall, 219 participants (219/303, 72.2%) completed the post survey (T2, 12 wk after baseline). The retention rate did not differ significantly between the IG (106/153, 69.3%) and the WCG (113/150, 75.3%; *χ*^2^_1_=1.39, *P*=.239). Non-completers (those who did not complete the post survey; mean 38.4, SD 11.62) were younger than those who completed the post survey (mean 41.57, SD 12.73, *t*_301_=2.00, *P*=.047). Regarding experience with psychotherapy before the study, noncompleters (number of previous psychotherapies: mean 2.67, SD 2.42) and completers (mean 3.75, SD 8.20) did not differ significantly (*t*_298_=1.18, *P*=.241). Additionally, no significant differences were found regarding gender (*χ*^2^_2_=3.08, *P*=.215), education level (*χ*^2^_4_=6.25, *P*=.181), or depressive symptom burden at the time of the baseline survey (BDI-II; dropouts mean 24.89, SD 9.85, completers: mean 27.07, SD 9.19; *t*_301_=1.81, *P*=.071).

### Intervention Adherence and Satisfaction

Participants in the IG logged in to the Novego program an average of 14 times (mean 14.88, SD 17.61) over the 12-week intervention period, with a range of 0 to 142. Of the 153 participants in the intervention group, 35 (22.8%) did not start the program. Satisfaction with the program, as measured by the CSQ-8, was on average 22.54 (SD 5.18), with 72.8% (75/103) of users saying they were either very or fairly satisfied with the program and 74.7% (77/103) saying they would recommend the program.

### Efficacy

#### Intention To Treat Analyses

For the primary endpoint (change in BDI-II at T2), the MMRM showed a significantly greater reduction in depressive symptoms in the IG compared to the WCG (see Table 2).

**Table 2.** Results of the ITT^a^ analyses with MMRM for the primary endpoint (BDI-II).

ITT	WCG	IG	Effect sizes and *P* values
Change from T0 to T1			LSMD^d^=2.65; Hedges *g=*0.36; *P*=.015
*n*^b^/*N*^c^ (%)	104/150 (69.3)	99/153 (64.7)	
Mean (SD)	−2.23 (7.66)	−5.15 (7.13)
LS Mean^e^ (SE)	−2.19 (0.75)	−4.84 (0.78)
Change from T0 to T2			LSMD=2.54; Hedges *g=*0.30; *P*=.017
*n*/*N* (%)	113/150 (75.3)	106/153 (69.3)	
Mean (SD)	−4.52 (8.31)	−6.91 (8.36)	
LS Mean (SE)	−4.51 (0.73)	−7.04 (0.76)	

a ITT: Intention-to-treat.

b *n*: Number of participants with baseline values in the analysis.

c *N*: Participants with baseline values.

d LSMD: Mean difference of least squares.

e LS Mean (SE): mean of leastsquarese.

Similarly, there was a significantly greater reduction in the secondary endpoint change in the sum score of PHQ-9 at T2 in the IG compared to the WCG (LSMD 1.65, 95% CI 0.71 to 2.59; *P*=.001, *g*=0.42).

#### Sensitivity Analyses

Using MI as the imputation method for the ITT sample, regression analyses confirmed a significantly greater reduction in depression observed in the IG compared to the WCG between T0 and T2, both in the BDI-II (*P*=.039, *g*=0.24) and in the PHQ-9 (*P*=.002, *g*=0.39). In the RSES, there was only a trend in favor of the IG (*P*=.073, *g*=0.22). No significant difference was found between the groups in the WHOQOL-BREF (*P*=.482, *g*=0.15). To further test the robustness of the ITT results for the primary endpoint, an additional ANCOVA using last observation carried forward as the imputation method was conducted. This analysis further confirms the results, showing a significantly greater reduction in depressive symptoms in the IG compared to the WCG (*F*_1;300_=4.68, *P*=.031, η_p_²=0.02).

In the CC population (WCG n=113, IG n=106), for the primary endpoint, a significantly greater reduction in depressive symptoms was observed in the IG, as measured by the BDI-II, compared to the control group (*F*_1;216_=5.51, *P*=.020, η_p_²=0.03; see Table 3). This result was confirmed by the PHQ-9, which also showed a significant group difference in the difference score between T0 and T2 (*F*_1;215_=9.88), *P*=.002, η_p_²=0.04; see Table 3). For the RSES, a significant improvement in self-esteem was observed in the IG compared to the WCG from T0 to T2 (*F*_1;213_=4.13, *P*=.043, η_p_²=0.02; see Table 3). However, for the WHOQOL-BREF, no significant difference was found (*F*_1;215_=0.55, *P*=.458, η_p_²=0.00; see Table 3).

**Table 3.** Mean values and standard deviations for CC, differences between the groups over time (ANCOVAs) for CC and PP.

	WCG^a^			IG^b^			ANCOVA^c^	
	Baseline, mean (SD)	Interim, mean (SD)	Post, mean (SD)	Baseline, mean (SD)	Interim, mean (SD)	Post, mean (SD)	CC^d^	PP^e^
Primary (T0–T2)								
BDI-II^f^	26.68 (9.80)	—^g^	22.31 (12.60)	27.49 (8.52)	—	20.45 (11.84)	*F*_1;216_=5.51, *P*=.020, η_p_^2^=0.03	*F*_1;193_=9.90, *P*=.002, η_p_^2^=0.05
Secondary (T0–T2)								
PHQ-9^h^	13.49 (5,22)	—	11.62 (5.58)	13.77 (4.85)	—	10.21 (5.29)	*F*_1;215_=9.88, *P*=.002, η_p_^2^=0.04	*F*_1;192_=15.20, *P*<.001, η_p_^2^=0.07
RSES^i^	14.34 (6.71)	—	15.14 (6.64)	13.50 (6.56)	—	15.68 (6.70)	*F*_1;213_=4.13, *P*=.043, η_p_^2^=0.02	*F*_1;193_=7.16, *P*=.008, η_p_^2^=0.04
WHOQOL^j^	41.29 (19.63)	—	46.88 (20.24)	38.09 (17.96)	—	46.70 (19.99)	*F*_1;215_=0.55, *P*=.458, η_p_^2^=0.00	*F*_1;192_=0.66, *P*=.418, η_p_^2^=0.00
Exploratory (T0–T1)								
BDI-II	—	2.10 (7.68)	—	—	5.26 (7.06)	—	*F*_1;201_=8.71, *P*=.004, η_p_^2^=0.04	*F*_1;184_=12.16, *P*<.001, η_p_^2^=0.06
PHQ-9	—	1.42 (3.54)	—	—	2.43 (3.46)	—	*F*_1;201_=2.82, *P*=.095, η_p_^2^=0.01	*F*_1;184_=4.21, *P*=.041, η_p_^2^=0.02

a WCG: waitlist control group.

b IG: intervention group.

c ANCOVA: analysis of covariance.

d CC: complete case sample.

e PP: per protocol sample.

f BDI-II: Beck Depression Inventory-II.

g Not applicable.

h PHQ-9: Patient Health Questionnaire-9.

i RSES: Rosenberg Self-Esteem Scale.

j WHOQOL: World Health Organization Quality of Life Short Questionnaire.

The improvement in depressive symptoms was already evident at the interim survey (6 wk after the initial survey). A significant difference in favor of the intervention group was also observed in the BDI-II (*F*_1;201=_8.71, *P*=.004, η_p_²=0.04). However, this was not confirmed in the PHQ-9 (*F*_1;201_=2.82, *P*=.095, η_p_²=0.01).

In the PP sample (WCG n=118, IG n=86), a significantly greater reduction in depressive symptoms was observed in the primary outcome in the IG, measured by the BDI-II, compared to the WCG (*F*_1;193_=9.90, *P*=.002, η_p_²=0.05; see Table 3). This result was confirmed by the secondary endpoint parameter, measured by the PHQ-9 (*F*_1;192_=15.20, *P*<.001, η_p_²=0.07; see Table 3). For the RSES, there was a significant improvement in self-esteem in the IG compared to the WCG from T0 to T2 (*F*_1;193_=7.16, *P*=.008, η_p_²=0.04; see Table 3). No significant group differences were observed for the WHOQOL-BREF scores (*F*_1;192_=0.66, *P*=.418, η_p_²=0.00; see Table 3).

### Negative Effects

In the postsurvey, the PANEPS-I assessed unintended positive and negative effects of the intervention. All IG participants with complete postsurveys were included in these analyses.

The frequencies of agreement per item for the positive and negative effects are shown in Tables4  5, respectively.

**Table 4.** Percentages of agreement of responses given (*n*) for the subscale positive effects. Missing values represent the choice of the response option 5 (“not applicable”). Percentages are valid percentages.

PANEPS-I^a^ items 1‐6	n	Agreement, n (%)
After completing the online program, I gained confidence in my own abilities.	82	44 (53.7)
I learned to take responsibility for myself during the online program.	83	49 (59.0)
My performance has improved as a result of the online program.	81	22 (27.2)
I am proud of myself for completing the online program.	76	42 (55.3)
Through the online program, I have been able to end stressful relationships.	70	16 (22.9)
Through the online program, my willingness to participate in face-to-face psychotherapy with a therapist has increased.	63	38 (60.3)

a PANEPS-I: Positive and Negative Effects of Psychotherapy Scale for Internet-Based Interventions.

**Table 5.** Numbers and percentages of agreement with statements regarding negative effects. Missing values represent the choice of the response option 5 (“not applicable”). Percentages are valid percentages.

PANEPS-I^a^ items 7‐27	n	Agreement n (%)
*Side effects*		
I am afraid that my social network will find out that I have been doing an online program.	98	4 (4.1)
The online program has worsened my relationship with my family and/or friends.	93	3 (3.2)
It bothers me that my family or friends treat me differently since I did the online program.	90	0 (0.0)
Since using the online program, I feel labeled as a mentally ill person.	91	8 (8.8)
My family or friends are embarrassed that I did an online program.	92	1 (1.1)
I am embarrassed that I have done an online program.	97	4 (4.1)
I’m afraid that I didn’t correctly implement certain exercises or understand the content and that, as a result, my condition has worsened.	93	14 (15.1)
*Malpractice*		
I experienced technical difficulties with the online program that bothered me.	93	11 (11.8)
The online program only worked towards “eliminating the problem”; there was no positive goal orientation.	72	9 (12.5)
The online program made me feel like I was to blame for my problems.	90	8 (8.9)
From my point of view, the applied therapeutic techniques of the online program were wrong.	77	6 (7.8)
The online program did not sufficiently address my personal problems.	84	35 (41.7)
I learned behaviors through the online program that were very harmful to me (eg, saying no excessively, setting myself apart).	87	7 (8.0)
The exercises and information in the online program did not match what my therapist or doctor told me.	72	4 (5.6)
I felt that my problems were not taken seriously in the online program.	87	10 (11.5)
I feel that I have changed for the worse as a result of the online program.	86	2 (2.3)
I did not feel addressed by the online program (eg, due to lack of gender-appropriate language or lack of customization).	86	12 (14.0)
The online program took too much time and/or put too much performance pressure on me.	94	46 (48.9)
*Unethical procedure*		
I felt pressured by the online program to do things I didn’t really want to do.	89	22 (24.7)
The online program seemed intolerant of my origin, religion, sexual orientation, or gender.	88	5 (5.7)
I had concerns about the privacy of my data.	94	5 (5.3)

a PANEPS-I: Positive and Negative Effects of Psychotherapy Scale for Internet-Based Interventions.

Over 60% (38/63) of the participants reported that their willingness to engage in conventional face-to-face therapy was increased due to the program. More than half reported increased confidence in their own abilities and responsibility for themselves.

At least one side effect was reported by 22.0% (22/100) of the participants (range 0‐5). Agreement with one or more items on the malpractice subscale gave 68.0% (66/97, range 0‐8), and one or more unethical procedure items were reported by 29.1% (29/97, range 0‐2). The most frequently reported negative effect was related to fear of incorrect implementation or understanding of the content and subsequent (subjective) deterioration (14/93, 15.1%). Regarding malpractice, 48.9% (46/94) said the program put too much time or performance pressure on the user and 41.7% (35/84) felt the program did not address personal problems sufficiently. An unethical procedure was reported by 24.7% (22/89) who felt pressured to do things they did not want to do.

Because of the significant age difference between completers and dropouts in this study, an exploratory analysis of age differences for the most frequently reported items of the PANEPS-I of each negative effect subscale was conducted using an independent *t*-test. For item 24 (“The online program took too much time and/or put too much performance pressure on me”) on the subscale, significantly younger participants (mean age 39.28, SD 11.47) reported this effect compared to those who did not agree with this item (mean age 47.65, SD 11.17; *t*_94_=3.63, *P*<.001). No significant difference in age was found for MP item 13 (“I’m afraid that I didn’t correctly implement certain exercises or understand the content and that, as a result, my condition has worsened”) on the side effects subscale (*t*_93_=−0.80, *P*=.429). There was a trend for younger age when agreeing (mean age 39.27, SD 13.00 vs mean age 44.93, SD 11.52) for item 25 (“I felt pressured by the online program to do things I didn’t really want to do”), but no significance was found (*P*=.055).

### Moderation Analyses

Moderation analyses were computed using the data from both the PP sample and the ITT sample, when applicable. The negative effects of the intervention (combined for the subscales of side effects, malpractice, and unethical procedure) had no significant moderating effect for the decrease in symptom severity in intervention users (PP, *P*=.184). Furthermore, treatment expectation did not significantly moderate the treatment effect in either the ITT sample (*P*=.489) or the PP sample (*P*=.773), and patient satisfaction did not significantly moderate the decrease in symptom severity in intervention users (PP, *P*=.896). Severity category according to the PHQ-9 did not demonstrate any significant interaction with the reduction in symptom burden (ITT: *P*=.984; PP: *P*=.967). In addition, for the ITT sample, experience with psychotherapy (dichotomized) independent of current treatment situation was evaluated as a moderator, but no interaction effect was found (*P*=.694). Effects of the moderation analyses are displayed in Table 6.

**Table 6.** Estimates (SE) of the moderation analyses in the per protocol and (where applicable) intention-to-treat samples.

Effect		Estimate	SE	*P v*alues
Per protocol				
#AE^a^				
(Intercept)		7.67	0.96	<.001
group		5.87	5.12	.255
#AE		0.01	0.01	.344
group*#AE		0.02	0.01	.184
TEX-Q^b^				
(Intercept)		5.97	0.60	<.001
group		3.57	1.22	.004
TEX-Q		0.85	0.54	.117
group*TEX-Q		0.31	1.08	.773
CSQ-8^c^				
(Intercept)		7.99	0.88	<.001
group		3.54	3.91	.368
CSQ-8		0.46	0.16	.005
group*CSQ-8		–0.11	0.86	.896
PHQ-9 category^d^				
(Intercept)		5.99	0.60	<.001
group		3.82	1.23	.002
PHQ-9 category		–0.27	0.77	.724
group*PHQ-9 category		0.06	1.57	.967
Intention-to-treat				
TEX-Q				
(Intercept)		4.59	0.78	<.001
group		2.24	1.12	.046
TEX-Q		0.48	0.83	.563
group*TEX-Q		0.78	1.12	.489
PHQ-9 category				
(Intercept)		5.42	2.20	.015
group		2.28	3.23	.480
PHQ-9 category		-0.40	0.97	.677
group*PHQ-9 category		0.03	1.40	.984
Psychotherapy experience				
(Intercept)		7.06	2.96	.018
group		0.67	4.18	.872
PT^e^ experience		–2.65	3.05	.386
group*PT experience		1.69	0.39	.694

a #AE: sum of reported adverse effects.

b TEX-Q: Treatment Expectation Questionnaire.

c CSQ-8: Client Satisfaction Questionnaire.

d PHQ-9: Patient Health Questionnaire-9.

e PT: psychotherapy.

## Discussion

### Principal Findings

Meta-analyses affirm the effectiveness of internet-based interventions for depression for alleviating symptoms and report advantages such as cost-effectiveness relative to CAU. However, not all users benefit, and negative effects can occur. Adverse events and side effects are largely neglected in research, which means that such costs for patients are scarcely considered. The aim of this two-arm randomized controlled trial was to investigate the effectiveness in terms of medical benefits of the internet-based program Novego Depression in adults with a depressive disorder and to assess negative effects occurring in users of the intervention.

The primary ITT analysis showed a significantly greater reduction in depressive symptom severity in the IG compared to the WCG after 12 weeks, both in the primary endpoint parameter (BDI-II) and in the secondary (PHQ-9), with small to medium effect sizes. The effect was already evident after 6 weeks. Sensitivity analyses (CC and PP) confirmed the results with greater effect sizes overall. For the secondary endpoints of self-esteem and quality of life, the hypothesized effects were not evident in the ITT analyses. However, in the CC and PP populations, a significantly greater improvement in self-esteem was found in the IG compared to the WCG.

The reduction of depressive symptoms is in line with previous findings on prior versions of the Novego Depression program with various patient groups [13-15]. The effect sizes are comparable to those found in meta-analyses with unguided interventions for depression (eg, [33]). The results of the present study now demonstrate that the intervention is effective in patients with a confirmed, primary diagnosis of depression who are already in contact with health care professionals because of their depression compared to waitlist controls with CAU. This emphasizes the benefits of this kind of interventions not only for those who do not want or cannot access face-to-face therapy but also for patients who want to “refresh” their therapy knowledge or want to have something to complement their ongoing therapy in daily life. Over 80% of participants experienced additional nonmedical positive effects when using the intervention, for example, gaining confidence or feeling proud of completing the online program.

Despite the positive effects of the intervention, users also experienced negative effects. Reported negative experiences were often related to concerns about using the program incorrectly and thus worsening one’s own condition or about feeling pressured by the program. The fact that side effects generally occur with online interventions is consistent with the results of other studies on adverse effects with similar interventions (eg, [17,19]). Usually, if adverse events are evaluated, the rate of symptom deterioration is reported. Karyotaki and colleagues [17] found in their meta-analysis that around 6% of internet-based intervention users experienced a worsening of symptoms. In this study, symptom deterioration was not evaluated, so no direct comparison can be made. However, 14.6% of the intervention users in this study reported concerns about worsening of symptoms due to their not implementing the psychotherapeutic methods properly. Other studies with a more qualitative evaluation of negative effects had comparable rates to the findings in this study (8%‐14% of users report side effects; [19,34]). The majority of the reported negative effects in those studies were program-related (eg, difficulty in understanding or implementing the exercises; 19), which is consistent with our results. Outliers with a higher rate in the current study were two items of the subscale MP (not sufficiently addressing personal problems and feeling pressured by the program), both with over 40%. When interpreting the results, it should be noted that these negative effects were assessed based on the purely subjective perspective of the users. Program-related negative effects like these might indicate a mismatch with the patient’s expectations or needs rather than actual malpractice. In addition, it must be mentioned that the questionnaire used for assessing negative effects only provides information about the occurrence of negative effects, not whether or to what extent these events actually had a negative, stressful, or damaging impact on the user.

Importantly, negative effects and other factors (expectation, satisfaction, and experience with psychotherapy) in this study did not moderate the change in depressive symptom severity. Therefore, although the frequency of these effects was not negligible, the experience of adverse effects during the intervention period did not exert a detrimental influence on the effect of the therapy. This also underlines the lack of assessing the valence or perceived extent of the reported negative effects. Fenski et al [19] found that experiencing negative effects with an internet-based intervention for depression predicts poor adherence, and Baumeister and colleagues [35] reported more program-related negative effects in non-responders among users of a digital self-help intervention for body-focused repetitive behaviors. Interestingly, in explorative analyses, age differences were found regarding reported negative effects. In line with the higher dropout rate in younger participants, those who reported feeling pressured by the program were significantly younger. These results further indicate that the acceptability of internet-based interventions might be dependent on age (and/or other patient characteristics).

### Limitations

The interpretative value of these results is compromised by some limitations that should be mentioned. The generalizability of this study is limited due to the predominantly female and well-educated sample and because all participants were German. Additionally, the results regarding negative effects of the intervention might be biased since only complete cases were analyzed. Missing data for the questionnaire used to assess negative effects (PANEPS-I) were not imputed. Further, the sample in this study was rather experienced with psychotherapy. This might also have an influence on the experience of possible negative effects. Less experienced users might feel more uncomfortable with challenging situations during the intervention and/or have different needs. Additionally, negative effects were assessed at the end of the 12-week intervention period. It is possible that the retrospective assessment distorted the participants’ answers. Because there was no follow-up assessment, no inference can be drawn regarding the sustainability of the treatment effects.

Lastly, as the questionnaire for assessing negative effects used in this study does not allow direct conclusions on the severity of the impact of experiencing these effects, a definite risk-benefit analysis cannot be conducted using the reported results. The moderation analysis solely indicates that the number of reported negative effects did not moderate the treatment effect.

It seems important to further investigate and consider negative effects in internet-based interventions as they can occur and their impact on (or cost to) the patient has not been well studied. Further research is needed to better understand the role of negative effects in internet-based interventions. Future studies on negative effects in internet-based interventions should also focus on investigating the extent and impact of potential negative effects and their moderating effect on treatment outcome and adherence. It is essential to further investigate how side effects arise and impact various subgroups of patients in order to achieve a better alignment between treatments and patients’ needs. This might help to further improve internet-based self-help interventions and better understand what works for whom.

### Conclusion

Overall, the Novego Depression program proved to be effective in reducing symptom severity compared to waitlist controls. Evaluation of negative effects showed that although they did occur, they did not reduce the treatment effect. Although some limitations must be considered when interpreting these results, it can be concluded that for participants who completed the surveys in this study, the digital health care application is an effective treatment and its benefits outweigh the reported negative effects.

## Supplementary material

10.2196/71274 Checklist 1 CONSORT-eHEALTH (Consolidated Standards of Reporting Trials of Electronic and Mobile Health Applications and Online Telehealth) checklist.

## Abbreviations

ANCOVA: analysis of covariance
BDI-II: Beck Depression Inventory II
CAU: care as usual
CBT: cognitive behavioral therapy
CC: complete case
CSQ-8: Client Satisfaction Questionnaire
iCBT: internet-based cognitive behavioral therapy
*ICD-10*: *International Statistical Classification of Diseases, Tenth Revision*
IG: intervention group
ITT: intention-to-treat
LSMD: least squares mean differences
MI: multiple imputation
MMRM: mixed-effects models for repeated measures
PANEPS-I: Positive and Negative Effects of Psychotherapy Scale for Internet-Based Interventions
PHQ-9: Patient Health Questionnaire-9
PP: per protocol
RSES: Rosenberg Self-Esteem Scale
SE: side effects
TEX-Q: Treatment Expectation Questionnaire
WCG: waitlist control group
WHOQOL-BREF: World Health Organization Quality of Life Brief
